# Organ-on-a-chip platforms for disease modeling and in vitro diagnostic applications

**DOI:** 10.3389/fbioe.2026.1956618

**Published:** 2026-09-17

**Authors:** Po Hao, Jingrong Deng, Jian Xu

**Affiliations:** 1 College of Medical Technology, Chongqing Three Gorges Medical College, Chongqing, China; 2 Research Center for Organs-on-Chips and Intelligent Detection, Chongqing Three Gorges Medical College, Chongqing, China

**Keywords:** biosensing, *in vitro* diagnostics, microfluidics, multi-organ integration, organ-on-a-chip

## Abstract

Organ-on-a-Chip (OoC) systems, as microphysiological models integrating microfluidic engineering, 3D tissue construction, and sensing technologies, can faithfully recapitulate the barrier functions, dynamic microenvironments, and inter-tissue crosstalk of human organs. They effectively address the inherent limitations of traditional 2D cell cultures and animal models in in vitro diagnostics (IVD), offering a novel avenue to tackle the high failure rate of candidate drugs and biomarkers in clinical translation. This review systematically summarizes the technological advances and application practices of OoC in the IVD field, covering core construction technologies (microfluidic regulation, cell and tissue engineering, material innovation), the integrated optimization of the “online real-time monitoring–offline in-depth analysis” dual-detection system, and typical applications in disease modeling, biomarker screening, drug toxicity evaluation, and personalized diagnosis. It further explores Multi-Organ-Chip (MOC) systems and Artificial Intelligence (AI) empowerment, analyzes core bottlenecks including insufficient physiological complexity, lack of standardization, and barriers to clinical translation and industrialization, and prospects future directions from technological optimization, standardization establishment, and regulatory pathway exploration. By focusing on the IVD-oriented perspective, this review aims to provide theoretical references and practical guidance for the transformation of OoC from laboratory research to clinical IVD tools.

## 1 Introduction

Traditional *in vitro* diagnostics (IVD) have long relied on 2D cell cultures, animal models, and endpoint detection methods. These systems possess inherent limitations in physiological relevance, dynamic response, and cross-species translatability, which constitute one of the key factors contributing to the persistently high failure rate of candidate drugs and biomarkers in clinical translation. Industry data indicate that only ∼10% of candidates receive final approval, while 90% fail during clinical development (Hay et al., 2014; Sun et al., 2022). Organ-on-a-Chip (OoC) integrates microfluidic engineering, 3D tissue construction, and cell biology technologies, enabling the recapitulation of organ-specific biomechanical stimuli, tissue interface functions, and dynamic fluidic environments at the microscale (Huh et al., 2010; Leung et al., 2022). Taking the classic “lung-on-a-chip” as an example, it can replicate human-like tissue functions and drug metabolism/toxicity responses *in vitro*, serving as a high-fidelity platform for disease modeling, drug safety evaluation, biomarker screening, and personalized diagnosis and treatment. This aligns with the developmental demands of IVD for “precision, dynamism, and individualization”. Meanwhile, regulatory and public funding agencies have continuously strengthened support for human-relevant alternative methods (NAMs). The U.S. Food and Drug Administration (FDA) and relevant evaluation bodies have successively issued policies to endorse the use of organoids and OoC in non-clinical safety and efficacy assessments, providing crucial policy support for the advancement of OoC toward clinically relevant IVD (Mirlohi et al., 2025; Dao and Sadrieh, 2026).

The development of OoC can be summarized along two complementary trajectories. One is the in-depth enhancement of biological simulation capabilities: from the early reconstruction of single-organ functions (e.g., the classic lung-on-a-chip simulating the alveolar-capillary interface and respiratory stretch) to multi-organ interconnected systems capable of maintaining mature tissue phenotypes and achieving physiological crosstalk between organs in recent years. The latter has demonstrated higher clinical relevance in studies on pharmacokinetics/pharmacodynamics (PK/PD) and inter-organ toxicity transmission mechanisms (Huh et al., 2010; Ronaldson-Bouchard et al., 2022). The other is the horizontal innovation of materials and detection methods: in terms of materials, traditional elastic polydimethylsiloxane (PDMS) is gradually being replaced by hydrogels (e.g., GelMA) and thermoplastic materials with lower small-molecule adsorption and enhanced mechanical-chemical tunability; in terms of detection, offline sampling and immunochemical detection are being replaced or supplemented by online *in situ* systems such as electrochemical biosensing, optical/fluorescent sensing, and microelectrode arrays (MEAs). This endows OoC with the capability of real-time, continuous, and multi-parameter collection of diagnostic information (Toepke and Beebe, 2006; Bupphathong et al., 2022; Ferrari et al., 2020). Notably, the hydrophobic small-molecule adsorption of PDMS and the influence of materials on cell-matrix interactions have promoted the research and development of biocompatible materials and standardized manufacturing processes. The second-generation technology replacing PDMS with bio-based membranes or soluble/degradable membranes has become a recent focus. Such improvements are directly related to the reproducibility and comparability of OoC in the quantitative evaluation of drugs and biomarkers (Toepke and Beebe, 2006; Zamprogno et al., 2021).

This review focuses on the IVD-oriented technological advances and application practices of OoC, excluding pure material research and non-diagnostic basic studies. It is structured around four core modules, progressing in a logical sequence: technological fundamentals (construction of single/multi-organ chips, material and microfluidic design, integration of sensing and detection systems), typical applications (disease modeling, preclinical toxicological evaluation, biomarker screening), intelligent empowerment (application and challenges of AI/machine learning in OoC data analysis and signal fusion), and bottlenecks and translation (model optimization, standardization construction, regulatory pathways and industrialization barriers, including the latest policy trends). This review aims to systematically sort out recent technological breakthroughs and clinically relevant cases, clarify the core technical and regulatory bottlenecks in the transformation of OoC as a humanized IVD platform from laboratory to clinic, and provide practical prospects and development path suggestions combined with the interdisciplinary integration trends of microfluidics, biomaterials, sensing, and AI. The subsequent sections will follow the logic of “technological fundamentals → typical applications → integrated intelligence → challenges and prospects”, providing references for researchers, clinical/regulatory personnel, and industrial decision-makers. Although several high-quality reviews have comprehensively covered OoC technologies from the perspectives of drug development and basic engineering principles (Leung et al., 2022; Ingber, 2022), these works focus primarily on pharmaceutical applications and platform design. By contrast, the present review is, to our knowledge, the first to systematically examine OoC specifically through the lens of *in vitro* diagnostics—covering diagnostic biomarker validation, sensor integration for clinical readouts, IVD-specific regulatory frameworks (including the FDA Modernization Act 2.0 and EU IVDR), and chip-to-clinic translation pathways—thereby complementing rather than duplicating existing reviews.

## 2 Core technological fundamentals of organ-on-a-chip and optimization for diagnostic compatibility

### 2.1 Core construction technologies and physiological microenvironment simulation

Organ-on-a-Chip (OoC) recapitulates the dynamic *in vivo* physiological microenvironment via the integration of microfluidic and cell engineering technologies. A classic example is the alveolus-on-a-chip, which features a dual-channel architecture: one channel perfused with flowing culture medium to mimic blood circulation, and the other with an air-liquid interface (ALI) exposed to air, with cyclic pneumatic stretching applied to simulate respiratory movements. This design not only provides continuous shear stress stimulation but also enables the epithelial-endothelial interface to maintain physiological functions under dynamic fluid forces (Stucki et al., 2015). Studies have demonstrated that fluid shear stress exerted through vascular channels can promote synchronized ciliary beating and mucociliary clearance, thereby enhancing the function of ciliated epithelium in respiratory tract models (Bai and Ingber, 2022). In addition, a simplified single-channel chip based on an induced pluripotent stem cell (iPSC)-derived airway progenitor cell culture platform has, for the first time, recapitulated the effects of fluid shear stress on airway ciliary development *in vitro*, and been applied to disease modeling of disorders such as primary ciliary dyskinesia (Roth et al., 2025).

Before proceeding, it is essential to clarify the conceptual relationship between organoids and organ-on-a-chip (OoC) platforms, as both terms appear throughout this review and are sometimes used interchangeably in the literature. Organoids are self-assembling, three-dimensional (3D) cellular structures derived from stem cells (pluripotent or adult) that recapitulate key architectural and functional features of their corresponding organs, including multiple cell types and tissue-level organization. However, organoids typically lack a controlled, dynamic microenvironment—they are usually cultured in static droplets or Matrigel domes without perfusion, mechanical stimuli, or integrated sensing capabilities. In contrast, OoC platforms are microfluidic devices engineered to provide precise control over the cellular microenvironment, including dynamic fluid flow (shear stress), mechanical strain (e.g., cyclic stretching for lung or heart chips), oxygen gradients, and real-time sensor integration, but they often rely on simpler cell configurations (e.g., 2D monolayers or basic co-cultures) rather than fully self-organized tissue structures. The convergence of these two technologies—termed “organoids-on-a-chip” or “chip-organoids”—combines the architectural fidelity of organoids with the controlled microenvironment and sensing capabilities of OoC, yielding platforms with enhanced physiological relevance for disease modeling and diagnostics (Reyes et al., 2024; Papamichail et al., 2025). A prominent application of this convergence is the Patient-Derived Organoid Chip (PDOC), in which patient-specific organoids are cultured within microfluidic chips to enable personalized drug sensitivity testing and biomarker discovery. For IVD applications, this distinction is critical: organoids alone provide structural biomimicry but limited dynamic readouts, whereas OoC platforms enable the continuous, multi-parameter monitoring required for diagnostic applications, and their integration offers the most promising path toward clinically relevant *in vitro* diagnostic models.

OoC enables the *in vitro* construction of complex tissues by combining stem cell technology with advanced techniques such as 3D bioprinting. For instance, a vascularized liver-on-a-chip fabricated via one-step 3D bioprinting uses polyvinyl acetate as the chip substrate, with cell-laden bioinks containing HepaRG hepatocytes and human umbilical vein endothelial cells (HUVECs) precisely bioprinted on its inner sides to form upper and lower dual channels (vascular lumen and biliary lumen) (Lee et al., 2019). This chip recapitulates the hepatic vascular-biliary system architecture, exhibiting more complete biliary secretory function and enhanced expression of liver function-related genes compared with biliary-lacking control models. In drug testing, the 3D liver-on-a-chip showed a more sensitive pharmacological response to acetaminophen (APAP) than 2D monolayer cultures, validating its reliability for simulating hepatic metabolism. Furthermore, sacrificial template-based bioprinting allows the fabrication of centimeter-scale liver-like tissues with branched vascular networks, where endothelial self-assembly forms microcapillaries and elevates the secretion of hepatocellular markers (e.g., albumin) (Liu et al., 2021). Overall, 3D bioprinting enables the high-precision construction of organ-specific vascularized models, facilitating the *in vitro* establishment of liver tissues with native structural and functional characteristics (Chae et al., 2023).

Polydimethylsiloxane (PDMS) is widely used for chip prototyping due to its biocompatibility and gas permeability, yet it suffers from drawbacks such as the strong adsorption of lipophilic drugs and non-protein molecules (Winkler and Herland, 2021). For example, the high surface area-to-volume ratio and organic surface adsorption of PDMS lead to unstable drug distribution; studies have shown that PDMS exhibits significantly higher adsorption loss of hydrophobic molecules (e.g., certain drug metabolites) compared with the thermoplastic cyclic olefin copolymer (COC) (Toepke and Beebe, 2006). Consequently, the industry has explored novel substrate materials: poly (ethylene oxide-co-vinyl acetate) (PEVA) has been used for fabricating liver-on-a-chip substrates (Carvalho et al., 2021); thermoplastic polymers with low autofluorescence and low adsorption (e.g., COC) for chip preparation, which reduces drug adsorption and yields more stable readouts (Ding et al., 2020; Mottet et al., 2014; van Midwoud et al., 2012). In addition, researchers have developed non-adsorptive silicones (e.g., PUMA, NOA81) and specialized hydrogels as alternatives, which retain the flexibility of PDMS while eliminating organic molecule adsorption (Sollier et al., 2011). For functional materials, the surfaces of some OoCs are modified via coating or integrated with functional components such as microelectrodes to meet specific sensing or biocompatibility requirements. For instance, silver/silver oxide electrodes integrated into hepatocyte culture modules enable the *in situ* monitoring of oxygen concentration and electrical conductivity (Wang Y. et al., 2024). In general, the diagnostic compatibility of OoC is improved through the optimization of material combinations and surface functionalization. The core construction technologies for physiological microenvironment simulation in representative alveolar-on-a-chip and liver-on-a-chip platforms, including microfluidic regulation, 3D bioprinted tissue fabrication and material optimization, are schematically illustrated in Figure 1, with the integrated sensing elements for diagnostic detection also depicted in detail. These construction technologies collectively establish the structural and functional foundation for OoC-based IVD platforms, enabling disease-relevant microenvironments that are prerequisite for generating reliable, clinically translatable diagnostic readouts.

**FIGURE 1:**
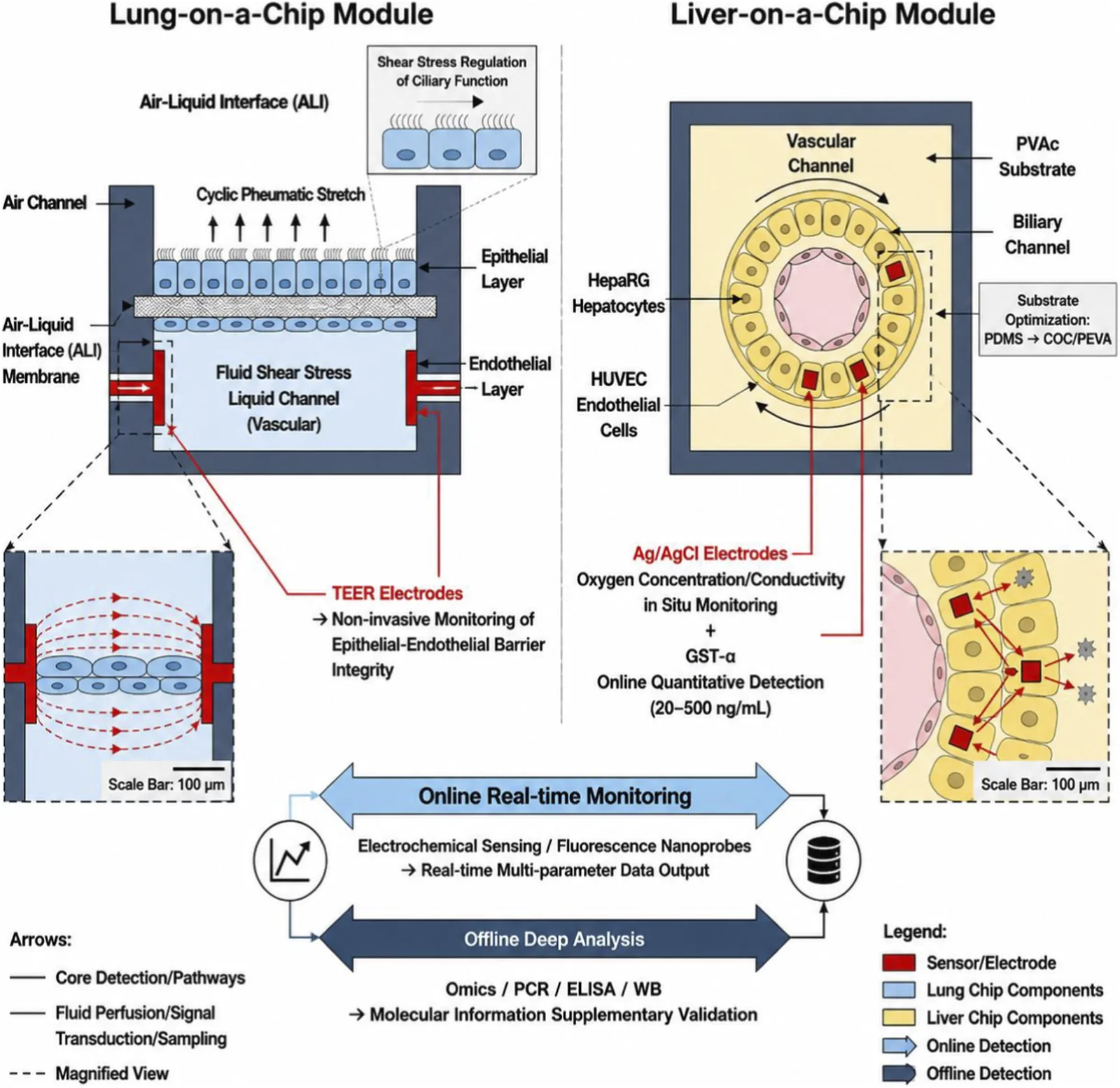
Schematic illustration of physiological microenvironment simulation and integrated detection systems for organ-on-a-chip platforms. The lung-on-a-chip module reconstructs the pulmonary microenvironment through a dual-channel microfluidic architecture, in which the air–liquid interface (ALI) supports airway epithelial cells and the vascular channel maintains endothelial cells under fluid shear stress. Cyclic pneumatic stretching mimics respiratory motion, while integrated TEER electrodes enable real-time monitoring of epithelial–endothelial barrier integrity. The liver-on-a-chip module incorporates HepaRG hepatocytes and HUVECs within a vascular–biliary microengineered structure, with 3D-printed PVAc substrates supporting hepatic tissue organization and Ag/AgCl electrodes enabling continuous monitoring of oxygen concentration and conductivity. The integrated GST-α detection system provides quantitative assessment of liver injury biomarkers. Overall, organ-on-a-chip platforms combine microfluidic regulation, mechanical stimulation, 3D tissue engineering, optimized biomaterials, and sensor integration to achieve online real-time monitoring through electrochemical sensing or fluorescence-based detection, followed by offline molecular validation using omics, PCR, ELISA, and Western blot analyses.

### 2.2 Integration of detection systems for in vitro diagnostic purposes

To achieve real-time monitoring and multi-dimensional diagnosis, organ-on-a-chip (OoC) integrates various online sensors, fluorescent nanoprobe technologies, and complementary offline analytical methods. In terms of online sensing, optical sensors (e.g., integrated miniature microscopes, optical fiber probes, or fluorescence detectors at the chip bottom) are applicable for real-time imaging and fluorescence signal detection (Ferrari et al., 2020). Electrochemical sensors (e.g., integrated redox electrodes, ion-selective electrodes, and microelectrode arrays) monitor metabolites (glucose, lactate, *etc.*) or cellular impedance/transmembrane conductance, and are commonly used to measure the Trans-Epithelial Electrical Resistance (TEER) of epithelial/endothelial barriers (Oliveira et al., 2021). Alveolus-on-a-chips integrate metal electrodes for TEER monitoring of the endothelial barrier, enabling non-invasive assessment of barrier integrity (Maoz et al., 2017; Henry et al., 2017). For mechanical sensing, silicone microcantilever arrays can be placed on the surface of cell culture chambers; the deflection of microcantilevers is recorded to estimate local forces (stress/elastic modulus) exerted by cells, providing readouts for myocardial contractility and the like (Morales et al., 2022). Additionally, flexible sensing elements based on strain-resistive or piezoresistive materials are utilized to measure the deformation of cell or tissue layers. The site-specific integration of electrochemical and optical sensors in alveolar-on-a-chip and liver-on-a-chip, as well as their corresponding diagnostic detection functions, are shown in Figure 1. In terms of quantitative performance, integrated electrochemical biosensors have achieved detection limits below 1 μM for dissolved oxygen with sensitivities of −0.58 μA·cm^-2^·μM^-1^ and stable operation over 8 days without measurable drift in protein-containing culture medium (Dornhof et al., 2022). Enzyme-based glucose and lactate biosensors reached limits of detection (LODs) of 7.6 μM and 6.1 μM, respectively, with highly linear and reversible responses across physiological concentration ranges (Dornhof et al., 2022). Multiplexed electrochemical immunobiosensors for albumin, GST-α, and creatine kinase MB (CK-MB) achieved ultralow LODs with sensitivities of 1.607, 1.105, and 1.483 log (ng·mL^-1^)^−1^, respectively, and wide dynamic detection ranges, enabling simultaneous multi-biomarker quantification on a single chip (Zhang et al., 2017). Optical pH sensors achieved sensitivities of 0.159 V·pH^-1^ over the range 6.5–8.0, and oxygen sensors showed sensitivities of 7 mV·O_2_%^−1^ with rapid response times, supporting continuous microenvironment monitoring for at least 5 days under fully automated operation (Zhang et al., 2017).

Recent advances have yielded quantitative performance benchmarks for integrated OoC sensors. Dornhof et al. (2022) developed a microfluidic organ-on-chip system with integrated electrochemical sensors for continuous multi-analyte monitoring of 3D cell cultures, achieving oxygen detection limits below 1 μM (sensitivity −0.58 μA·cm^-2^·μM^-1^), glucose LOD of 7.6 μM, and lactate LOD of 6.1 μM, with stable continuous operation exceeding 8 days without signal drift and a measurement interval of 2 min (Dornhof et al., 2022). In parallel, Aleman et al. demonstrated regeneratable electrochemical affinity biosensors capable of monitoring up to 3 biomarkers per sampling-detection cycle of approximately 1 h, with functionalization and regeneration requiring ∼7 h, enabling continual biomarker tracking on OoC platforms (Aleman et al., 2021). These quantitative benchmarks establish performance baselines for the analytical validation of OoC-based diagnostic sensors.

Fluorescent and nanoprobe technologies serve as another category of common online detection tools. For example, the calcium-sensitive dye Fluo-4 AM can be added to cardiomyocyte culture media to monitor intracellular Ca^2+^ transient concentration changes, thereby evaluating the electrical activity and contractile function of cardiomyocytes (Gourisaria et al., 2021). Nanoprobes such as quantum dots (QDs) possess high photostability and multi-labeling capability, making them suitable for real-time labeling of cellular components or detection of specific molecules. One study utilized flexible elastomers doped with cadmium selenide (CdSe)/zinc sulfide (ZnS) core-shell QDs in *ex vivo* heart-on-a-chip systems, enabling simultaneous electrical stimulation and real-time fluorescent contraction monitoring (Wu et al., 2024). Another work developed a liver-on-a-chip hydrogel system doped with graphene quantum dots, realizing online quantitative detection of α-glutathione S-transferase (GST-α) secreted by hepatocytes with a linear detection range of 20–500 ng/mL and high sensitivity and selectivity (Wang Y. et al., 2024). Furthermore, upconversion nanoparticles (UCNPs) and other nanoprobes have been applied in microphysiological systems (MPS)/OoC platforms to characterize inter-tissue interfaces, cellular transport phenomena, and targeted imaging. These nano-optical probes achieve visual tracking of molecular events in the microenvironment via near-infrared (NIR) excitation and multi-color emission (Wang et al., 2026).

To complement online monitoring, offline detection is often employed as a supplementary method. At the end of the experiment or specific time points, cells and culture media in the chip can be sampled for analysis, including omics analyses (transcriptomics, proteomics, metabolomics, *etc.*), PCR/qPCR for gene expression detection, Western Blot for protein analysis, and ELISA/substrate colorimetric assays for secreted biomarkers (Chen et al., 2022). For instance, ELISA can be used to detect kidney injury marker KIM-1 and inflammatory factor IL-6 in kidney-on-a-chip culture media (Banaeiyan et al., 2017; Yu et al., 2024; Chethikkattuveli Salih et al., 2022); Western Blot and qPCR are applicable to verify changes in tight junction proteins, cytokines, and signaling pathways expressed in chip tissue layers (Liu et al., 2023). Although offline methods require sample destruction, they provide supplementary molecular information that is difficult to obtain directly via online sensing, establishing a comprehensive “online + offline” diagnostic system. This dual-detection architecture constitutes the analytical core of OoC-based IVD, bridging real-time physiological monitoring with clinical-grade molecular analysis to support diagnostic decision-making. These personalized chip platforms represent a paradigm shift in IVD, transitioning from population-based reference ranges to individual-specific functional diagnostics that can directly inform clinical treatment selection.

## 3 Typical application scenarios of organ-on-a-chip in in vitro diagnostics

### 3.1 Disease modeling and early diagnosis

Organ-on-a-Chip (OoC) has been widely used to construct *in vitro* disease models and assist in early diagnosis. For infectious diseases, lung tissue chips are employed to simulate respiratory viral infection processes. For example, a biomimetic alveolus-on-a-chip developed by Cao et al. was used to study SARS-CoV-2 infection, recapitulating the mucociliary-immune barrier of human alveolar epithelium/endothelium and evaluating the efficacy of antiviral drugs (Cao et al., 2022). They found that drugs such as probenecid (PPS) could significantly inhibit viral spread on the chip. In addition, by simulating pathogen-host interactions, OoC has been applied to investigate the infection mechanisms of pathogens such as influenza virus and *Mycobacterium tuberculosis* (Thacker et al., 2020).

For metabolic and barrier-related diseases, liver chips are used to study lipid metabolism disorders (e.g., non-alcoholic fatty liver disease, NAFLD), and can recapitulate pathological features such as lipid accumulation in patients with NAFLD *in vitro* (Deguchi and Takayama, 2022). Wang et al. developed a hiPSC-derived liver organoid-on-a-chip system that modeled NAFLD by exposing organoids to free fatty acids, resulting in lipid droplet accumulation, upregulated lipid metabolism genes, and increased reactive oxygen species (ROS) alongside inflammatory and fibrogenic markers, providing a quantifiable disease phenotype platform for diagnostic biomarker discovery (Wang et al., 2020). Similarly, Wiriyakulsit et al. established a HepG2-based steatosis liver-on-a-chip that exhibited declined viability and function under lipid overload, enabling efficacy and toxicity evaluation of therapeutic candidates while simultaneously serving as a diagnostic model for monitoring steatosis progression markers (Wiriyakulsit et al., 2023). Meanwhile, blood-brain barrier (BBB)-on-a-chip and gut-liver cascade chips are utilized to simulate BBB function, providing *in vitro* models for neurodegenerative diseases or inflammatory bowel diseases (Cui and Cho, 2022). Mir et al. reviewed how biosensor-integrated BBB-on-chip systems enable real-time monitoring of barrier integrity and neurodegenerative disease progression markers, offering a dynamic diagnostic platform that complements static post-mortem tissue analysis (Mir et al., 2022). Kempuraj et al. further summarized recent progress in brain-on-a-chip and BBB-on-a-chip models for neurodegenerative disorders, highlighting integrated microelectrode arrays and real-time readouts that enhance diagnostic throughput for screening neurodegenerative therapeutics and assessing barrier function (Kempuraj et al., 2026).

Currently, no specific reports have been dedicated to islet chips for diabetes diagnosis, but islet organoid chips have shown potential for drug screening and physiological research (Yin et al., 2022). In tumor diagnosis, tumor chips are used to analyze the sensitivity of patient tumor tissues to chemotherapeutic drugs. In this field, “Patient-Derived Organoid Chips (PDOC)” can predict chemotherapeutic responses *in vitro*. For instance, Hu et al. developed an integrated superhydrophobic microwell array chip (InSMAR-chip) for high-throughput culture and analysis of lung cancer organoids (LCOs). Using organoids generated from patient surgical and biopsy samples, the platform predicted patient-specific drug responses within 1 week, achieving 100% accuracy and specificity when compared with clinical outcomes. The on-chip drug sensitivity results were in good agreement with patient-derived xenograft models, genetic mutations, and actual clinical treatment responses, demonstrating the diagnostic potential of PDOC for rapid functional drug response testing (Hu et al., 2021). Additionally, microfluidic tumor chips are employed to capture circulating tumor cells (CTCs) for early diagnosis. Cai et al. developed and clinically validated a microfluidic-based CTC enrichment platform that achieved 87.1% recovery rate, approximately 4-log depletion of white blood cells, and 95.10% detection accuracy across 572 cancer patients. The platform processed 4 mL of whole blood within 15 min, with ROC analysis yielding an AUC of 0.9267 for distinguishing cancer patients from healthy subjects. At the optimal cutoff of 4 CTCs/4 mL, sensitivity was 80.77% and specificity was 95.18%, substantially outperforming conventional immunomagnetic capture methods (<40% capture efficiency) (Cai et al., 2023). A review indicated that microfluidic structures integrated into chips can efficiently enrich CTCs in blood, holding promise for early tumor screening (Kashaninejad et al., 2016). More recently, Yuan et al. demonstrated a deep learning-powered scalable cancer organ chip platform that combines high-throughput patient-relevant drug testing with AI-driven image analysis, offering a functional precision oncology framework for cancer diagnosis and treatment selection (Yuan et al., 2026). Regmi et al. further reviewed the broad applications of microfluidics and OoC in cancer research, noting advances in CTC isolation, tumor microenvironment modeling, and drug response profiling that collectively enhance cancer diagnostic capabilities (Regmi et al., 2022).

Further advancing tumor diagnostic applications, Steinberg et al. developed a fully 3D-printed versatile tumor-on-a-chip that enables multi-drug screening and demonstrated correlation between on-chip drug responses and actual clinical patient outcomes, establishing the platform as a functional diagnostic assay for personalized treatment selection (Steinberg et al., 2023). Wang et al. reviewed OoC applications in cancer therapy and reported that patient-derived organoids cultured on chips achieved over 87% drug-response accuracy in colorectal cancer, highlighting the potential of OoC-based functional diagnostics for precision oncology treatment selection (Wang et al., 2025).

Compared with existing IVD technologies, OoC-based disease models offer several complementary advantages. First, they provide dynamic, time-resolved monitoring of disease progression rather than static endpoint snapshots, enabling the capture of transient diagnostic biomarkers that may be missed by conventional assays. Second, OoC platforms support functional diagnostics—assessing not merely the presence of a biomarker but its functional consequence in a physiologically relevant context, as exemplified by drug response testing on PDOC platforms. Third, patient-derived OoC models enable individual-specific diagnostic readouts, moving beyond population-based reference ranges toward personalized diagnostic thresholds. Fourth, the multi-parametric, multi-sensor integration on OoC platforms allows simultaneous monitoring of multiple biomarkers (e.g., barrier integrity, metabolic activity, inflammatory cytokines, and morphological changes), providing a more comprehensive diagnostic profile than single-analyte conventional assays. These attributes position OoC as a complementary diagnostic modality that fills gaps in the current IVD landscape, particularly for complex, multifactorial diseases where dynamic and functional assessments are diagnostically valuable (Ingber, 2022; Regmi et al., 2022).

### 3.2 Screening and validation of diagnostic biomarkers

The development of diagnostic biomarkers on OoC platforms can be systematically organized along the biomarker development pipeline—from discovery through preliminary validation, confirmatory validation, and ultimately clinical translation—as conceptualized by the FDA-NIH Biomarkers, EndpointS, and other Tools (BEST) framework (Califf, 2018; Kraus, 2018). This staged framework provides a structured approach to evaluating the diagnostic value of OoC-derived biomarkers and clarifying the distinct role of OoC at each stage.

At the discovery stage, OoC platforms serve as high-fidelity discovery engines by recapitulating disease-relevant microenvironments that elicit physiologically authentic biomarker release. For instance, in COVID-19 research, significantly elevated levels of cytokines IL-6 and IL-8 secreted by epithelial and immune cells in pneumonia or inflammation model chips were identified as candidate indicators for monitoring infection or inflammation status (Cao et al., 2022). In a pilot proteomics study, Millet et al. used mass-spectrometry-based analysis of a human-derived microvascular microfluidic lumen to identify 35 candidate biomarkers potentially predictive of ionizing radiation exposure, demonstrating the utility of OoC platforms for biomarker discovery in a controlled, physiologically relevant context (Millet et al., 2021). This systematic, stage-wise framework—from discovery through clinical translation—distinguishes OoC’s dual role in biomarker research: as a discovery platform that generates candidate biomarkers under physiologically relevant conditions, and as a validation platform that benchmarks these biomarkers against clinical standards. This dual capability is unique to OoC and distinguishes it from conventional biomarker research tools, positioning it as an integrated platform for the entire biomarker development pipeline in IVD applications.

At the confirmatory validation stage, OoC platforms must be benchmarked against established clinical standards to demonstrate predictive validity. A paradigmatic example is the systematic evaluation of the human Liver-Chip by Ewart et al., who tested 870 chips across 27 hepatotoxic and non-toxic drugs and demonstrated 87% sensitivity and 100% specificity for predicting drug-induced liver injury—meeting the qualification guidelines of the Innovation and Quality consortium and substantially outperforming animal models and 3D spheroid cultures (Ewart et al., 2022). This study represents the first large-scale, quantitative validation of an OoC platform against clinical outcomes, establishing a benchmark for confirmatory validation of OoC-based diagnostic biomarkers.

At the clinical translation stage, OoC-derived biomarkers must be integrated into clinical diagnostic workflows. Jenkins et al. recently explored the translation of OoC technology for human-relevant diagnostic biomarkers, emphasizing the need to bridge the gap between OoC-generated biomarker data and clinical diagnostic applications (Jenkins et al., 2026). The BEST framework provides the regulatory vocabulary and categorization (diagnostic, monitoring, pharmacodynamic/response, predictive, prognostic, safety, and susceptibility/risk biomarkers) necessary for classifying OoC-derived biomarkers according to their intended clinical use (Califf, 2018; Kraus, 2018). Successful clinical translation requires not only analytical validation but also clinical validation and, where appropriate, regulatory qualification—processes that demand collaboration between OoC developers, clinical laboratories, and regulatory authorities. This staged framework highlights that OoC’s value to IVD extends across the entire biomarker development pipeline, from discovery through to clinical translation, positioning OoC as a versatile platform technology for next-generation diagnostics.

In liver and kidney disease models, multi-sensor technologies have been used to dynamically monitor biomarkers such as GST-α and alcohol dehydrogenase secreted by the liver, as well as KIM-1 and IL-6 released by the kidney. On liver chips, a combination of fluorescent probes and biochemical analyses is adopted to real-time record enzymatic secretion levels of GST-α and other enzymes. On kidney chips, ELISA is used for quantitative detection of KIM-1 and IL-6 to reflect the degree of renal tubular injury (Chethikkattuveli Salih et al., 2022). By configuring multiple sensing elements in a single chip, simultaneous monitoring of multiple biomarkers (e.g., lactate, glucose, and cytokines in culture media) can be achieved, thereby establishing a multi-dimensional dynamic diagnostic indicator system (Oliveira et al., 2021). Notably, a multisensor-integrated platform demonstrated continuous *in situ* monitoring for at least 5 days with fully automated operation, detecting dose-dependent changes in albumin (decreasing) and GST-α (increasing) secretion upon acetaminophen treatment at 0, 5, and 10 mM doses, with results correlating with endpoint cell viability assays (Zhang et al., 2017). These quantitative readouts illustrate the potential of OoC-integrated biosensing to generate clinically relevant, dynamic biomarker profiles that static endpoint assays cannot capture.

### 3.3 Companion diagnostics and pharmacodiagnostic applications

While organ-on-a-chip (OoC) platforms have been extensively employed for preclinical drug toxicity and efficacy screening, their diagnostic relevance lies in the emerging paradigm of companion diagnostics (CDx) and pharmacodiagnostics—using patient-specific drug response data generated on OoC platforms to guide clinical treatment decisions and patient stratification (Ingber, 2022; Peck et al., 2020). Companion diagnostics are essential for identifying patients likely to benefit from a specific therapeutic agent, and OoC platforms offer a unique functional CDx approach by testing patient-derived cells against therapeutic candidates *ex vivo* under physiologically relevant conditions.

A landmark demonstration of OoC as a predictive diagnostic tool was provided by Ewart et al., who systematically evaluated a commercial human Liver-Chip across 27 hepatotoxic and non-toxic drugs. The Liver-Chip achieved 87% sensitivity and 100% specificity in predicting drug-induced liver injury (DILI), substantially outperforming both animal models and 3D hepatic spheroid cultures. This level of diagnostic performance met the qualification guidelines established by the Innovation and Quality consortium, representing the first systematic, quantitative benchmarking of an OoC platform against clinical toxicity outcomes (Ewart et al., 2022). Such predictive capability directly supports diagnostic workflows in which chip-based drug response data inform clinical decision-making regarding drug safety and patient risk stratification.

Beyond toxicity prediction, OoC-based pharmacodiagnostic platforms enable functional testing of patient-specific drug responses. Patient-derived organoid-on-a-chip (PDOC) systems can predict individual chemotherapeutic responses within clinically actionable timeframes, effectively serving as functional diagnostic assays that complement genomic biomarker testing. This approach transforms drug response data from a preclinical screening endpoint into a diagnostic readout that directly informs treatment selection (Ingber, 2022). Peck et al. argued that by 2030, OoC systems could become integral to clinical pharmacology, enabling patient-specific predictions of drug efficacy and safety that inform diagnosis, treatment selection, and dosing within the clinical workflow (Peck et al., 2020). The integration of OoC-derived drug response data with clinical databases further enables the construction of “chip-clinical phenotype” prediction models, upgrading from passive diagnostic classification to active therapeutic prediction (Zhou et al., 2025). In this context, OoC-based drug response testing serves not merely as a preclinical tool but as a functional diagnostic platform that bridges the gap between *in vitro* pharmacology and clinical diagnostic decision-making, particularly for companion diagnostic applications in precision oncology and personalized pharmacotherapy. This functional diagnostic approach represents a distinct IVD application that complements traditional molecular biomarker-based companion diagnostics. These pharmacodiagnostic capabilities position OoC as a complementary diagnostic modality that bridges functional drug response testing with companion diagnostic development, enabling biomarker-driven patient stratification through physiologically relevant *in vitro* assays.

### 3.4 Personalized in vitro diagnostics and precision medicine

Constructing personalized chip platforms using patient-derived cells is a promising direction in precision medicine. Researchers have utilized patient-specific induced pluripotent stem cell (iPSC)-derived cardiomyocytes to build heart chips, simulating patient-specific genetic heart disease models. These cells retain the patient’s genetic background, enabling *in vitro* screening of personalized drug efficacy and toxicity (Deir et al., 2024; Tang et al., 2022). For instance, heart chips constructed with patient cells can test individual responses to different antiarrhythmic drugs, realizing “personalized drug identification via the chip”.

Similarly, studies have attempted to generate islet organoids from iPSCs of diabetic patients and culture them long-term on chips to evaluate patient-specific islet function (Liu et al., 2020). A review indicated that human iPSC (hiPSC)-derived organoids can serve as platforms to recapitulate key phenotypes of metabolic disorders such as phenylketonuria (PKU), offering a new approach for studying metabolic abnormality mechanisms and potential drug intervention strategies. This strategy is expected to further enhance the physiological relevance of models by integrating organoids into microfluidic OoC systems (Bor et al., 2021; Zhu et al., 2026). These personalized chip platforms represent a paradigm shift in IVD, transitioning from population-based reference ranges to individual-specific functional diagnostics that can directly inform clinical treatment selection. The representative organ-on-a-chip platforms discussed across these application scenarios are summarized in Table 1.

**TABLE 1:** Summary of representative organ-on-a-chip platforms for IVD applications.

Organ system	Cell source	Integrated sensors	Validated applications	Key Refs
Lung	Alveolar epithelial/endothelial cells	TEER	SARS-CoV-2 infection modeling, antiviral efficacy evaluation	(Stucki et al., 2015; Maoz et al., 2017; Henry et al., 2017; Cao et al., 2022; Thacker et al., 2020)
Liver	HepaRG/HepG2 hepatocytes; hiPSC-derived hepatocytes; HUVECs	Electrochemical (O_2_), fluorescent probes, biochemical assays	NAFLD/steatosis modeling, DILI prediction	(Lee et al., 2019; Wang Q. et al., 2024; Wang et al., 2020; Wiriyakulsit et al., 2023; Ewart et al., 2022)
Heart	iPSC-derived cardiomyocytes	Calcium imaging, microcantilever, ECG	Cardiotoxicity screening, arrhythmia detection	(Morales et al., 2022; Gourisaria et al., 2021; Wu et al., 2024; Deir et al., 2024; Tang et al., 2022)
Kidney	Primary renal tubular cells	ELISA	Nephrotoxicity assessment, KIM-1/IL-6 monitoring	(Chethikkattuveli Salih et al., 2022)
BBB	Brain endothelial cells, pericytes, astrocytes	Biosensors, microelectrode arrays	Neurodegenerative disease modeling, barrier integrity monitoring	(Cui and Cho, 2022; Mir et al., 2022; Kempuraj et al., 2026)
Gut	Intestinal epithelial cells	Biochemical assays	Drug absorption and toxicity modeling	(Yu et al., 2024)
Tumor	Patient-derived organoids; CTCs	Microfluidic capture, imaging	Drug response prediction, CTC enrichment	(Hu et al., 2021; Cai et al., 2023; Kashaninejad et al., 2016; Steinberg et al., 2023)
Multi-organ	Multiple organ-specific cells	Multi-modal (optical, electrochemical, mechanical)	Systemic PK/PD, inter-organ crosstalk	(Edington et al., 2018; Novak et al., 2020; Picollet-D’hahan et al., 2021)

## 4 Technological integration and innovation: multi-organ integration and AI empowerment

### 4.1 Multi-Organ-Chip systems

Multi-Organ-Chip (MOC) systems achieve connection and information exchange between different organ models via interconnection technologies such as circulating culture media. They overcome the limitation of single-organ chips in capturing inter-tissue interactions, providing a more comprehensive *in vitro* platform for complex disease diagnosis and systemic toxicity evaluation. Edington et al. developed 4-, 7-, and 10-organ connected microphysiological systems, which can maintain stable cell function across multiple chips in a continuous circulation system for weeks and support pharmacokinetic studies (Edington et al., 2018). Novak et al. used robotic fluidic interconnects to link 8 vascularized dual-channel organ chips (intestine, liver, kidney, heart, lung, skin, blood-brain barrier, and brain), maintaining organ-specific functions for over 3 weeks in a “blood-like” common medium (Novak et al., 2020). A recent review indicated that MOC can simulate systemic disease processes by supporting inter-organ information exchange, and is gradually integrating neural and immune systems to enhance physiological relevance (Picollet-D’hahan et al., 2021). These multi-organ integrated platforms can real-time recapitulate pathophysiological coupling effects between organs, such as co-culturing central nervous and immune cells to simulate neuro-inflammatory crosstalk, providing more reliable in vivo-like models for complex disease diagnosis and systemic toxicity prediction. In particular, immune-competent OoC platforms have emerged as a critical frontier. Lymph node-on-a-chip models recreate the lymph node microenvironment with stromal cells, dendritic cells, and T/B lymphocytes in multi-compartment architectures, enabling *in vitro* studies of immune cell activation, antigen presentation, and vaccine responses (Shanti et al., 2020; Wang Q. et al., 2024). Bone marrow-on-a-chip systems with vascularized niches have sustained CD34^+^ hematopoietic stem/progenitor cell maintenance and demonstrated functional neutrophil egress in response to G-CSF and doxorubicin, providing a platform for studying hematopoietic toxicity and myeloid cell dynamics (Glaser et al., 2022). More broadly, immunocompetent organ chips that integrate circulating immune cells (e.g., macrophages, neutrophils, T cells) with tissue-specific models enable the study of immune–tissue crosstalk under inflammatory, infectious, and oncological conditions (Morrison et al., 2024). These immune-integrated platforms are essential for disease modeling (infection, autoimmunity, cancer) and for discovering immune-related diagnostic biomarkers such as dynamic cytokine profiles and immune cell phenotypic shifts, which are difficult to capture in conventional static cultures. The multi-organ PK/PD data generated on MOC platforms can further support pharmacodiagnostic applications by informing individualized dosing biomarkers, bridging systemic drug response prediction with clinical diagnostic decision-making for personalized therapy optimization.

### 4.2 AI empowerment

With the advancement of multi-sensor technologies, Artificial Intelligence (AI) has begun to empower OoC-based diagnostics. Chip platforms can integrate multi-modal sensors (optical, electrochemical, mechanical, *etc.*) to realize real-time label-free monitoring of cell behaviors and drug responses (Yang et al., 2024). AI algorithms can automatically extract phenotypic features and patterns from these large-scale datasets. For example, deep learning-based microscopic image recognition has been applied to cell classification on lung chips. After supervised learning training on hundreds of microscopic images, the classifier achieved an AUC accuracy of over 95%, effectively distinguishing different cell types and quality states in the chip (George and Kenry, 2025). AI can also optimize experimental conditions and data processing workflows: studies have shown that AI-driven visual recognition and data analysis tools are applicable for culture condition optimization, image detection and tracking, and large-scale data processing (Deng et al., 2023; Yin et al., 2026). Furthermore, data obtained from chips can be linked to clinical databases to construct “chip-clinical phenotype” prediction models, upgrading from passive diagnosis to active prediction and accelerating the clinical translation of chip-based diagnostic models (Zhou et al., 2025).

Beyond image classification, specific AI/ML algorithms are being tailored to the diverse data modalities generated by OoC platforms. Convolutional neural networks (CNNs) have become the workhorse for morphological analysis of chip-cultured cells: a review by Anderson and Sarmadi highlighted that CNN-based approaches—particularly when combined with transfer learning and data augmentation—can significantly improve OoC image analysis performance on small datasets, enabling accurate cell trajectory tracking, high-resolution segmentation, and robust classification with limited labeled images (Anderson and Sarmadi, 2024). In a proof-of-concept study, Tak et al. integrated a 3D microfluidic bladder cancer organ-on-a-chip with CNN-based image analysis to predict anticancer drug resistance. Using a dataset of 2,674 cell images with data augmentation and a step-decay learning rate, the CNN achieved 95.2% accuracy, average sensitivity of 90.5%, specificity of 96.8%, and AUC values exceeding 0.988 for classifying four levels of gemcitabine resistance, demonstrating the diagnostic potential of AI-integrated OoC for functional drug response prediction (Tak et al., 2023).

For time-series sensor data generated by OoC biosensors—including electrochemical, mechanical, and optical readouts—recurrent neural networks (RNNs) and Transformer models offer powerful analytical frameworks. RNN-based methods have been extensively reviewed for computational physiology applications, demonstrating effectiveness in analyzing and predicting temporal physiological processes such as cardiac rhythm dynamics, barrier function fluctuations, and metabolic oscillations (Mao and Sejdic, 2023). Transformer architectures, with their self-attention mechanisms, are particularly suited for modeling long-range temporal dependencies in multi-sensor OoC data streams, enabling early detection of subtle phenotypic shifts that may precede overt diagnostic endpoints.

Physics-informed neural networks (PINNs) represent an emerging approach for integrating mechanistic PK/PD models with OoC-generated data. By embedding physical governing equations directly into the neural network loss function, PINNs can model drug absorption, distribution, metabolism, and excretion processes on multi-organ chips while respecting known pharmacokinetic constraints. Ahmadi et al. reviewed the broad application of physics-informed machine learning in biomedical science and engineering, highlighting its potential for bridging data-driven and mechanistic modeling paradigms in complex biological systems including OoC platforms (Ahmadi et al., 2026). This approach is particularly valuable for diagnostic applications where mechanistic interpretability of model predictions is essential for clinical acceptance.

A persistent challenge for AI integration in OoC-based diagnostics is the scarcity of large, annotated datasets. Unlike clinical imaging or genomics, OoC data are typically generated in small batches with significant inter-experiment variability. To address this, transfer learning strategies have been explored: Kenneweg et al. demonstrated that Siamese networks combined with synthetic data generation can effectively overcome small-sample limitations in microfluidic single-cell cultivation analysis (Kenneweg et al., 2023). Movčana et al. contributed by releasing an OoC image dataset of 3,072 cell images specifically designed for training machine learning classifiers, providing a shared resource to accelerate AI model development in the OoC community (Movčana et al., 2024). Chen et al. showed that label-free imaging combined with deep learning and transfer learning can accurately predict the differentiability of human small airway epithelial cells from day-3 images, enabling early quality assessment of airway-on-chip models with minimal training data (Chen et al., 2024). A comprehensive overview by Li et al. further summarized the growing intersection of deep learning and OoC technologies, emphasizing that data augmentation, transfer learning, and multi-modal data fusion are critical strategies for overcoming the inherent data scarcity in OoC-based AI applications (Li et al., 2022).

The concept of digital twins—virtual replicas of physical systems—is increasingly being integrated with OoC platforms to create predictive diagnostic models. Gangwal and Lavecchia reviewed how AI-enhanced digital twins combined with OoC can enable precise simulations of complex biological systems, improving predictive power and scalability for drug safety evaluation (Gangwal and Lavecchia, 2025). Roy and Cucullo further outlined an integrated framework in which OoC readouts (barrier integrity, biomarkers, electrophysiology, and multi-omics) are converted into time-resolved quantitative signals that can be benchmarked against patient-level data, enabling AI-enabled, cross-platform digital twin analyses for translational decision-making (Roy and Cucullo, 2026). In this paradigm, the OoC platform serves as the physical sensor layer while the digital twin provides the computational inference layer, together forming a closed-loop diagnostic system capable of real-time prediction and personalized clinical recommendation.

As AI-assisted OoC diagnostics approach clinical deployment, model interpretability and regulatory considerations become paramount. Kim et al. reviewed the critical importance of transparency in medical AI systems, emphasizing that explainable AI (XAI) methods—such as SHAP (Shapley Additive Explanations) and LIME (Local Interpretable Model-agnostic Explanations) — are essential for building clinician trust and enabling regulatory approval (Kim et al., 2026). However, a systematic analysis by Mehta et al. revealed pervasive transparency gaps in FDA-reviewed AI/ML medical devices, with an average AI Characteristics Transparency Reporting score of only 3.3 out of 17, and nearly half of devices not reporting any performance metrics even after FDA guidance (Mehta et al., 2025). For OoC-based AI diagnostics to achieve regulatory approval as IVD devices, developers must proactively address these transparency requirements, including clear documentation of training data, model architecture, validation metrics, and decision boundaries. The regulatory pathway will likely involve the FDA’s Software as a Medical Device (SaMD) framework, which requires a Total Product Lifecycle approach addressing algorithmic bias, safety, and equity—considerations that are particularly challenging for OoC-based AI given the heterogeneity of biological data and the novelty of the platform (Mehta et al., 2025). The convergence of multi-organ integration and AI empowerment is thus transforming OoC from a research tool into a clinically actionable diagnostic platform capable of supporting complex diagnostic and prognostic decisions.

## 5 Existing challenges and technical bottlenecks

### 5.1 Physiological complexity limitations

The physiological complexity of current OoC models remains insufficient, and some key systems have not been fully integrated. For instance, most models lack the coordination of neuro-immune-endocrine systems, failing to fully recapitulate the real multi-system crosstalk in the human body (Picollet-D’hahan et al., 2021). In addition, heterogeneity of cell sources severely affects model consistency: commonly used commercial cell lines are easy to handle but have low physiological relevance, while induced pluripotent stem cells (iPSCs) or primary cells are more human-relevant but difficult to culture and usually exhibit immature phenotypes (Caballero et al., 2022). Poor long-term culture stability is another major issue. Currently, most multi-organ systems can maintain *in vitro* functions for only a few weeks; prolonged culture often leads to drift in cell phenotype and function (Edington et al., 2018; Zhao et al., 2019). Collectively, these factors result in deviations between some current chip models and human physiology, limiting their reliability. A comprehensive review pointed out that human OoC still cannot meet the requirements of clinical predictability and reliability, while the use of transplantable models and independent offline quality control can significantly improve system stability and reproducibility (Renggli et al., 2019). Although immune-competent OoC models—including lymph node-on-a-chip, bone marrow-on-a-chip, and immune cell co-culture chips—are emerging as promising platforms, most current OoC systems still lack full immune system integration. Key challenges include maintaining primary immune cell viability and function in long-term microfluidic culture, recapitulating the complex architecture of secondary lymphoid organs, and standardizing immune cell sourcing (donor variability, cryopreservation effects) across laboratories (Ingber, 2022; Morrison et al., 2024). Without integrated immune components, OoC models cannot fully capture the inflammatory cascades and immune-mediated tissue damage that underpin many diagnostic biomarker signatures, limiting their translational relevance for immune-related diseases.

### 5.2 Lack of standardization

There is a lack of unified standards for OoC fabrication processes. Commonly used polymer materials such as PDMS exhibit drawbacks of small-molecule drug adsorption and significant batch-to-batch variation, impairing data consistency. Meanwhile, no uniform specifications exist across laboratories regarding material ratios, channel design, or culture medium components, hindering cross-institutional comparison and validation of results. Simultaneous multi-parameter detection also poses a technical bottleneck: existing sensing technologies struggle to achieve high spatiotemporal resolution detection of multiple physical quantities within a single chip, necessitating the development of highly sensitive, label-free integrated sensing solutions. Additionally, incompatibility between chip data quantification and clinical diagnostic standards limits practical application—most current data remain as raw laboratory results, which are difficult to directly align with clinical reference values (Leung et al., 2022). Thus, widespread adoption of OoC hinges on enhanced industrial standardization, including unified guidelines for chip design, fabrication, and data processing.

### 5.3 Commercialization and regulatory barriers

Despite its potential advantages, OoC suffers from high commercialization costs and complex operational workflows, failing to meet the high-throughput demands of clinical diagnostics. For example, expensive materials, microfabrication equipment, and stringent requirements for automated multi-channel fluid control systems render commercial chips costly. Meanwhile, the regulatory pathway for OoC-based diagnostic technologies remains unclear: existing regulations primarily target traditional IVD and biological agents, lacking dedicated approval criteria for novel diagnostic reagents based on microphysiological systems, which slows technical dissemination (Skardal, 2024; Ma et al., 2021). Analyses indicate that although OoC and organoid platforms have attracted regulatory attention, their formal incorporation into approval processes requires addressing challenges such as large-scale data processing and reproducibility (Skardal, 2024). Collectively, high commercial costs, operational complexity, and ambiguous regulatory requirements constitute major barriers to OoC industrialization. Addressing these challenges is essential for OoC to transition from proof-of-concept studies to validated IVD tools that meet clinical laboratory standards for accuracy, reproducibility, and regulatory compliance.

Recent legislative and regulatory developments have begun to address these barriers. The FDA Modernization Act 2.0, signed into law on 29 December 2022, removes the statutory mandate for animal testing in drug development and redefines “nonclinical tests” to explicitly include *in vitro*, *in silico*, and microphysiological systems (MPS), providing a legislative foundation for the regulatory acceptance of organ-on-a-chip (OoC) technologies (Han, 2023; Zushin et al., 2023).

In September 2024, FDA accepted Emulate’s Liver-Chip S1 into the Innovative Science and Methods Approaches (ISTAND) pilot program—marking the first OoC technology accepted as a drug development tool for predicting drug-induced liver injury (DILI) in Investigational New Drug (IND) submissions. The ISTAND qualification process follows a three-step pathway: a letter of intent (LOI), a qualification plan, and a full qualification package. Furthermore, FDA’s April 2025 roadmap outlines a plan to make animal studies “the exception rather than the norm” within 3–5 years, signaling a systemic shift toward human-relevant *in vitro* platforms (Ingber, 2026).

Standardization efforts are also gaining momentum. ISO 22916:2022 provides guidelines for microfluidic device interoperability, applicable to OoC design and fabrication. The NIST-led Organ-on-a-Chip Engineering Standards Working Group—comprising FDA, EU regulatory bodies, academia, and industry representatives—has published a framework for MPS standardization, with initial focus on heart, kidney, and liver models (Reyes et al., 2024). Additionally, the IQ MPS Consortium’s pharmaceutical industry survey indicates growing MPS adoption in drug development, though challenges remain in regulatory acceptance and cross-site reproducibility (Baker et al., 2024).

Collectively, a practical regulatory pathway for OoC-based IVD products is emerging: analytical validation (demonstrating accuracy, precision, and analytical sensitivity) followed by clinical validation (establishing clinical performance against a reference standard), and regulatory submission via FDA 510(k) or *De Novo* pathway for IVDs, or the ISTAND pathway for drug development tools; in the EU, conformity assessment under the IVDR is required. The development of OoC-specific performance standards—including standardized cell sources, sensor calibration protocols, and benchmark reference materials—remains a critical need for regulatory harmonization (Reyes et al., 2024; Ingber, 2026).

## 6 Future prospects and development directions

### 6.1 Advanced materials and sensor innovation

Future research will focus on advancing novel chip materials and components to enhance model physiological relevance. On one hand, biodegradable polymers (e.g., POMaC, GelMA) and stimuli-responsive hydrogels can create a more body-compatible microenvironment while avoiding long-term residues. On the other hand, integrating high-sensitivity, single-cell resolution sensors (e.g., electrochemical biosensors, nanospectroscopic sensors) will enable label-free real-time detection. Furthermore, integrating systemic systems such as neural-immune networks is a key direction: ongoing studies aim to reconstruct complex circuits like the gut-brain axis and blood-brain barrier immune crosstalk on chips, facilitating more accurate simulation of systemic disease processes.

### 6.2 Standardization and high-throughput development

To meet clinical needs, the coordinated development of industrial standards and high-throughput technologies is imperative. Academic and industrial communities should jointly establish unified standards for chip design, fabrication, and quality control to ensure inter-equipment consistency and reproducibility. Building on this, high-throughput OoC platforms based on standardized modular design can be developed, integrating automated pipetting robots and parallel analysis systems to enable mass chip production and parallel operation. Current studies have attempted robotic automated culture and high-speed microfluidic distribution systems to maintain interconnected multi-organ chips for weeks—this platform integrates automated culture, fluidic connection, and real-time imaging, enabling coordinated culture and analysis of up to eight vascularized organ chips under standard conditions for over 3 weeks (Novak et al., 2020). The recent 384-well plate-based chip (e.g., IFlowPlate) further demonstrates the feasibility of high-throughput detection in microphysiological simulations (Ma et al., 2021). These innovations will significantly improve chip detection throughput, making it more suitable for large-volume clinical samples.

### 6.3 Chip-clinical validation system

To truly serve clinical practice, a rigorous “chip-clinical” validation system must be established to confirm the correlation between chip model results and human phenotypes. On one hand, clinical sample comparison trials should be conducted to verify the chip’s ability to predict patient disease phenotypes or drug responses. Meanwhile, collaboration with regulatory authorities (e.g., FDA, EMA) is essential to clarify approval processes and standards for OoC-based diagnostic technologies. In the future, diagnostic chip kits for specific diseases (e.g., tumor biomarker chips) are expected to emerge, accelerating the translation from laboratory research to clinical application.

### 6.4 Interdisciplinary integration and closed-loop diagnostics

Looking ahead, efforts should be directed at strengthening the in-depth integration of organ-on-a-chip (OoC) with artificial intelligence (AI), synthetic biology, and 3D bioprinting, to build an integrated closed-loop system from simulation to diagnosis and treatment. In terms of AI, it can be applied to intelligent analysis of chip data, parameter optimization, and result prediction. Synthetic biology enables the construction of programmable cellular circuits on chips, realizing dynamic regulation and feedback. 3D bioprinting, meanwhile, supports high-precision fabrication of complex tissue structures, providing a more natural 3D microenvironment for chips. In the future, the integration of these interdisciplinary technologies will drive the formation of a “simulation-detection-prediction-diagnosis-treatment” closed loop. For instance, spatiotemporal omics microfluidic chips can simultaneously acquire large-scale omics information at both tissue and cellular levels. The continuous integration of new technologies will lead a paradigm shift in in vitro diagnostics (IVD), moving from traditional sample-based detection to a new microphysiology-based system. These future directions collectively chart a path toward OoC becoming a mainstream IVD modality, integrated into clinical laboratory workflows and diagnostic decision-making alongside traditional assay platforms.

## 7 Conclusion

Organ-on-a-Chip (OoC) technology has demonstrated remarkable potential in disease modeling, biomarker screening, companion diagnostics, and personalized medicine by recapitulating human organ-level physiology with integrated, real-time sensing capabilities. As the field transitions from proof-of-concept demonstrations toward clinical diagnostic applications, three core bottlenecks must be urgently addressed. First, the absence of unified standardization—encompassing chip fabrication protocols, cell sourcing criteria, sensor calibration procedures, and data reporting formats—impedes inter-laboratory reproducibility and regulatory review. Second, insufficient clinical validation: the majority of OoC platforms remain at the proof-of-concept stage, and large-scale, multi-center clinical studies benchmarking chip-derived readouts against established diagnostic gold standards remain scarce. Third, scalability and cost barriers: current fabrication and operational workflows are incompatible with the high-throughput, low-cost demands of routine clinical diagnostics.

Objectively, the technical roadmap toward OoC-based IVD products will likely unfold in phases. In the near term (3–5 years), OoC platforms are expected to gain regulatory traction as drug development tools—exemplified by FDA ISTAND acceptance of Emulate’s Liver-Chip—and to serve as complementary diagnostic assays in companion diagnostic and pharmacodiagnostic applications. Medium-term milestones (5–10 years) will require concerted progress in ISO/NIST-led standardization, automated high-throughput manufacturing, and prospective clinical validation studies. Full market entry of OoC-based standalone IVD products for routine clinical use is realistically achievable within 10–15 years, contingent on sustained investment and regulatory clarity.

To accelerate this trajectory, targeted actions are needed from three stakeholder groups. Funders should prioritize sustained support for clinical validation studies, standardization initiatives, and cross-institutional data-sharing consortia that generate the evidence base required for regulatory qualification. Regulators must develop fit-for-purpose review pathways for OoC-based IVD devices—building on the FDA Modernization Act 2.0, ISTAND, and EU IVDR frameworks—with clear analytical and clinical validation criteria specific to microphysiological systems. Industry should invest in scalable manufacturing (e.g., injection-molded thermoplastic chips), automated workflow integration, and standardized quality control to bridge the gap between prototyping and clinical-grade production. Through coordinated industry-academia-regulatory collaboration, OoC technologies can mature into a reliable, clinically validated diagnostic modality, realizing a new microphysiology-based paradigm for precision medicine.

## Glossary

OoC: Organ-on-a-Chip
IVD: *In Vitro* Diagnostics
MOC: Multi-Organ-Chip
MPS: Microphysiological Systems
PDMS: Polydimethylsiloxane
COC: Cyclic Olefin Copolymer
TEER: Transepithelial Electrical Resistance
MEA: Microelectrode Array
ALI: Air-Liquid Interface;
iPSC: Induced Pluripotent Stem Cell;
hiPSC: Human Induced Pluripotent Stem Cell
PDOC: Patient-Derived Organoid-on-a-Chip
CTC: Circulating Tumor Cell
CDx: Companion Diagnostics
DILI: Drug-Induced Liver Injury
BBB: Blood-Brain Barrier
NAFLD: Non-Alcoholic Fatty Liver Disease
ROS: Reactive Oxygen Species
PK/PD: Pharmacokinetics/Pharmacodynamics
AI: Artificial Intelligence
ML: Machine Learning
CNN: Convolutional Neural Network
RNN: Recurrent Neural Network
XAI: Explainable Artificial Intelligence
ISTAND: Innovative Science and Methods Approaches for New Drugs
IVDR: *In Vitro* Diagnostic Regulation
NIST: National Institute of Standards and Technology
ISO: International Organization for Standardization
QDs: Quantum Dots; ELISA: Enzyme-Linked Immunosorbent Assay
PCR: Polymerase Chain Reaction
ECM: Extracellular Matrix
3D: Three-Dimensional
2D: Two-Dimensional
